# Salivary MMP-9 as a Biomarker for the Diagnosis of Oral Potentially Malignant Disorders and Oral Squamous Cell Carcinoma

**DOI:** 10.31557/APJCP.2020.21.1.233

**Published:** 2020

**Authors:** Komal Smriti, Meenakshi Ray, Tilottama Chatterjee, Revathi-Panduranga Shenoy, Srikanth Gadicherla, Kalyana-Chakravarthy Pentapati, Nasrullah Rustaqi

**Affiliations:** 1 *Department of Oral Medicine and Radiology, *; 4 *Department of Oral and Maxillofacial Surgery, *; 5 *Department of Public Health Dentistry, Manipal College of Dental Sciences, *; 3 *Department of Biochemistry, Kasturba Medical College, Manipal, Manipal Academy of Higher Education, Manipal, Karnataka, *; 2 *Radiant Eye foundation, Elgin Road, Kolkata, India, *; 6 *Department of Oral and maxillofacial surgery, Dentistry faculty of Kabul Medical University, Kabul, Afghanistan. *

**Keywords:** MMP, saliva, noninvasive, biomarker, cancer

## Abstract

**Objective:**

To compare the salivary MMP – 9 concentration among subjects with oral squamous cell carcinoma (OSCC), oral potentially malignant disorders (OPMD), tobacco users, and control groups.

**Materials and methods:**

A total of 88 subjects were enrolled and divided into four study groups viz., OSCC (n=24), OPMD (n=20), tobacco habits (n=22), and healthy controls (n=22). All subjects gave unstimulated saliva samples for the evaluation MMP – 9 by ELISA kit. Demographic information like age, gender, type of tobacco, and duration of the habit were recorded.

**Results:**

Subjects with OSCC and OPMD had significantly higher mean MMP-9 levels than subjects with tobacco habits and control groups (P<0.001). Also, poorly differentiated OSCC group had significantly higher mean saliva MMP-9 than moderate and well-differentiated OSCC. The optimal cut-off point was 214.37 ng/mL with a sensitivity of 100% and specificity of 59% for OSCC versus the control group. The optimal cut-off point was as 205.87 ng/mL with a sensitivity of 100% and a specificity of 54% for OPMD versus the control group.

**Conclusion:**

The data obtained from this study indicated that OSCC and OPMD had an increased level of salivary MMP-9. Salivary MMP-9 could be a useful, non-invasive adjunct technique in the diagnosis, treatment, and follow-up of OSCC and OPMD.

## Introduction

Oral squamous cell carcinoma (OSCC) is one of the most prevalent cancers in the world, with a high incidence and a low survival rate of 50-60% in developing countries. (Warnakulasuriya, 2009; Seki et al., 2011; Ghallab and Shaker, 2017) OSCC is often diagnosed late with metastatic changes at the initial screening due to its asymptomatic nature in the early stages. (Hamada et al., 2012) The delay in identification also occurs due to a lack of early detection tools for potentially malignant and OSCC lesions. A biopsy followed by histopathological examination is still the gold standard. Also, choosing an accurate site for biopsy can be challenging because of the irregular appearance of oral potentially malignant disorders (OPMD) and OSCC. “WHO defines OPMD as clinical presentations that carry a risk of cancer development in the oral cavity, whether in a clinically definable precursor lesion or in clinically normal oral mucosa”. Conditions like leukoplakia, erythroplakia, erythroleukoplakia, oral submucous fibrosis (OSMF), palatal lesion of reverse cigar smoking, and oral lichen planus are considered as OPMD (Soares et al., 2018). OSCC and OPMD are associated with multiple risk factors like the use of tobacco, areca nut, alcohol, exposure to chemicals, immunosuppression, diet, viral infections, hormones, and sunlight (Blatt et al., 2016). The current treatment modality is based on long-established staging indices (TNM criteria) and histopathological assessment. A wide array of diagnostic markers are explored in the past as an alternative to conventional invasive methods (drawing blood or biopsy). Salivary biomarkers offer a promising diagnostic adjunct due to its simple non-invasive collection method. 

Saliva has been used in novel ways to diagnose various oral and systemic conditions. It is used for glucose estimation for detection of diabetes,(Smriti et al., 2016) cortisol levels to determine stress levels, (Gadicherla et al., 2018) various cytokines and tumor markers for diagnosis of OSCC, (Gutiérrez-Corrales et al., 2017; Deepthi et al., 2019) detection of infections like HIV(Holm-Hansen et al., 2007) and identification of blood groups(Takizawa et al., 1989), cotinine (Honarmand et al., 2018) etc. Salivary diagnostics has evolved as a dynamic field over the last decade and serves as a part of molecular diagnostics. The non-invasive, inexpensive collection method and easy storage of saliva have made it a feasible modality to predict the diagnosis (Dineshkumar et al., 2016; Nosratzehi et al., 2017; Seyedmajidi et al., 2018) and outcome of oral neoplasms, as well as monitoring the post-therapy prognosis of OSCC (Gutiérrez-Corrales et al., 2017). This could be possible as most biomarkers present in serum and urine can also be traced in saliva. 

MMPs function as mediators of alterations of carcinogenesis. They may also have a role in the regulation of initial steps of carcinogenesis and invasion (Yadav et al., 2014). Among the numerous salivary biomarkers, Matrix Metalloproteinase-9 (MMP-9) has gained much attention because it causes proteolytic disintegration of the basement membrane by directly targeting meshwork of type IV collagen as well as collagens V, VII and X, fibronectin and elastin. This is a critical step that facilitates tumor invasion and metastasis. 

The proximity of saliva with the oral lesion helps it sustain detectable levels of MMP-9 and hence aids in diagnosis (Wu et al., 2010; Venugopal and Uma Maheswari, 2016). Serum (Mardani et al., 2014; Lotfi et al., 2015) and tissue MMP-9 (Katayama et al., 2004) has been studied previously, while studies on salivary MMP-9 are scant. A recent meta-analysis (Hema Shree et al., 2019) reported only one study that compared the salivary MMP-9 among OSCC, OPMD, and controls. (Ghallab and Shaker, 2017) Considering the role of MMP-9 on the incidence and progression of OPMD and OSCC, we aimed to compare the salivary levels of MMP-9 among subjects with OSCC, OPMD, tobacco habits, and healthy control group.

## Materials and Methods

We conducted a cross-sectional comparative study among four groups of subjects enrolled from the departments of Oral Medicine and Radiology and Oral and Maxillofacial Surgery at our institution. The study included 88 subjects divided into four study groups viz., OSCC (n=24), OPMD (n=20), tobacco habits (n=22) and healthy controls (n=22). Kasturba Hospital and Kasturba Medical College Institutional Ethics Committee approved the study protocol (898/2017), and the same was registered with the clinical trial registry of India (CTRI/2018/01/011566). Informed consent was sought from all the subjects before the commencement of the study.

*Sample size calculation*

The sample size calculation was done using MedCalc Statistical Software version 14.8.1 (MedCalc Software, Ostend, Belgium). With a power of 90% and Alpha of 0.05, a sample of 17 subjects per group would be required for an area under the curve (AUC) 0.8 to be significant when compared to the null hypothesis of 0.5. 

*Saliva Sample collection*

Unstimulated saliva samples were obtained from all the subjects in the morning. Subjects were instructed not to eat, brush their teeth, or use mouth rinse at least 2 hours before sample collection. Subjects were then instructed to swallow and make chewing motions for 30 seconds and were told to tilt the head forward and expectorate 5 mL of saliva into a sterile container. 

*Sample collection for Biopsy*

A biopsy followed by histopathologic examination confirmed the diagnosis of OSCC and OPMD. Demographic information like age, gender, mode of tobacco consumption, frequency, and duration of the habit were recorded. The data sheet included clinical parameters like site, size, staging according to TNM classification and histological grading of tumor.

*Inclusion criteria*

Histopathologically confirmed cases of OSCC and OPMD subjects aged 18 and above, and those consented to participate were included in the study. OPMD group had subjects with leukoplakia, erythroplakia and OSMF. Tobacco habits group included subjects who consumed tobacco in any form for more than one year without any sign of clinical OSCC or OPMD. The control group included healthy individuals with no tobacco habit and other comorbidities. 

*Exclusion criteria*

Subjects with autoimmune diseases, previous history of malignancy, prior treatment in the form of chemotherapy, radiotherapy, surgery or alternative medicine, immunodeficiency, autoimmune disorders, hepatitis, or human immunodeficiency virus infections, pregnancy or lactation were excluded.

*Estimation of MMP-9*

The samples were transported on ice to the laboratory immediately for processing. The dilution ratio of 1:50 was considered to assay all the samples and was re-run at 1:100 if the values were outside the standard curve. Each sample was clarified by centrifu¬ging at 4000g at 4°C for 10 mins and stored. The MMP-9 levels were measured in the thawed samples using Human MMP-9 PicokineTM ELISA kit (Boster Biological Technology, Pleasanton, CA, USA). The kit implemented the sandwich ELISA technique, wherein the MMP-9 in the sample binds to the MMP-9 specific polyclonal antibody coated on the microplate. A secondary biotinylated MMP-9 specific antibody binds to the MMP-9 bound to the plate and facilitates detection. The intensity of the resultant mixture was read at 450 nm. A set of standards were run alongside the samples, and the final values were obtained in ng/mL. The procedure was performed by a trained and calibrated technician. 

*Statistical analysis*

All the analysis was done using SPSS version 18 (PASW Statistics for Windows, Version 18.0. Chicago: SPSS Inc). A p-value of <0.05 was considered statistically significant. ANOVA with post-hoc Games Howell test or Tukey’s test was used for inter-group comparisons. Receiver Operating Characteristic (ROC) curve was plotted to evaluate the cut-off points of saliva MMP-9. 

## Results

A total of 88 subjects were included; the majority of them were males (n=62) with a mean age of 51.23±14.06 years (Table 2). The majority of the subjects in the OSCC group had a histopathological diagnosis of squamous cell carcinoma (n=22). Two subjects in the OSCC group had verrucous carcinoma. Among the OPMD group, the conditions included were erythroplakia (n=8), OSMF (n=7), and leukoplakia (n=5) (Table 1). 

The mean saliva MMP-9 concentration was significantly different among the four study groups (P<0.001). The post-hoc test showed that subjects in OSCC and OPMD groups had significantly higher mean saliva MMP-9 than subjects in tobacco habits and control groups. There was no significant difference between OSCC and OPMD groups nor tobacco habits and controls (Table 3). A sub-group analysis showed a significant difference in the mean saliva MMP-9 levels among the three stages of OSCC (P=0.014). Post-hoc analysis showed that the mean saliva MMP-9 levels in subjects with poorly differentiated OSCC was significantly higher than moderate and well-differentiated OSCC. No significant difference was seen in the mean MMP-9 levels among the different conditions of OPMD (P=0.128) (Table 4). 

**Figure 1:**
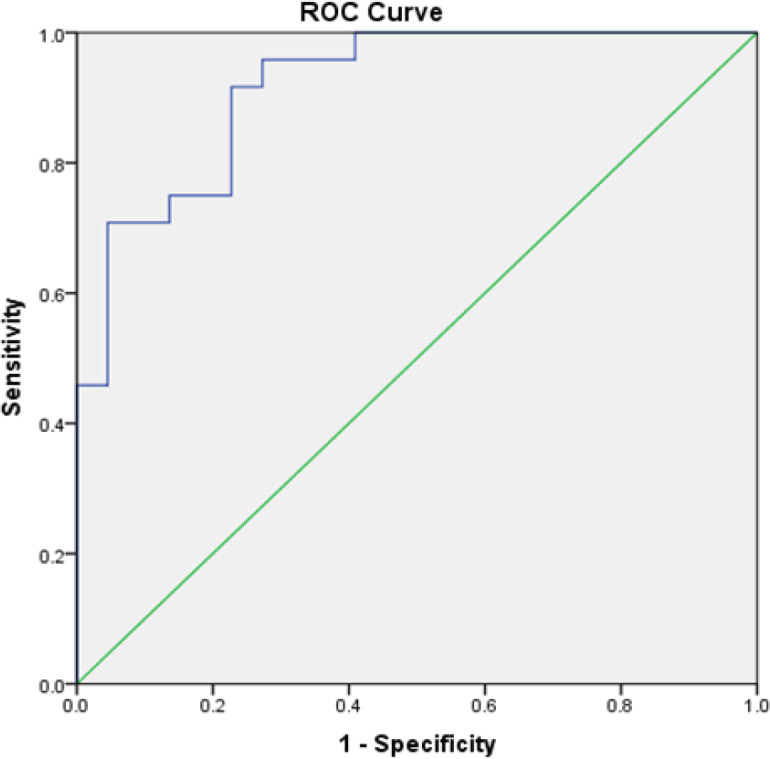
ROC Curve of Salivary MMP-9 in the Diagnosis OSCC for OSCC versus Healthy Controls

**Figure 2:**
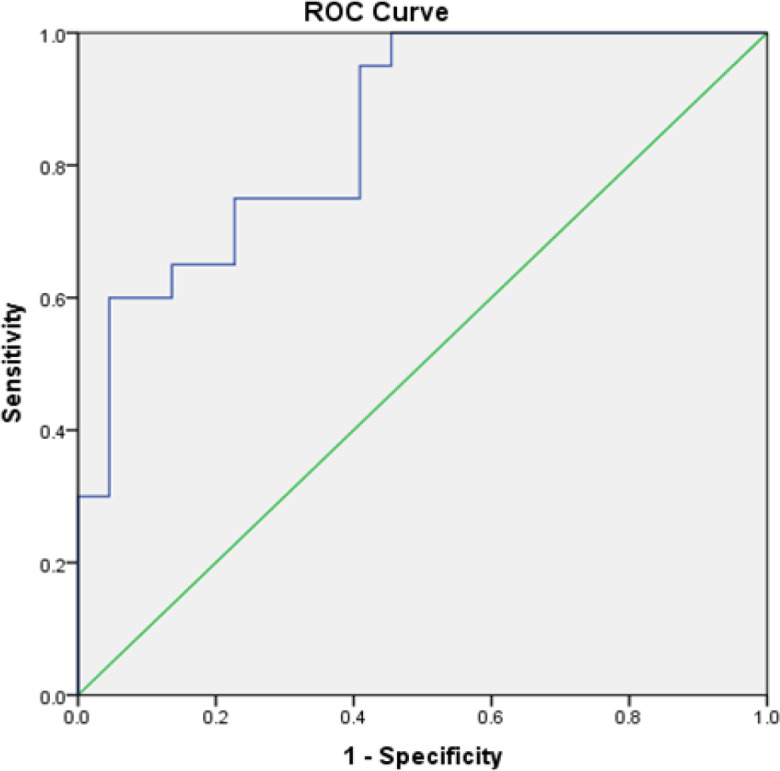
ROC Curve of Salivary MMP-9 in the Diagnosis OPMD for OPMD versus Healthy Controls

Two ROC curves were plotted viz., OSCC group versus the control group, and OPMD group versus the control group. The Area under the curve (AUC) for OSCC versus the control group was 0.917 (95% CI: 0.84-0.99) (Figure 1). The optimal cut-off point was 214.37 ng/mL with a sensitivity of 100% and a specificity of 59%. The AUC for OPMD versus the control group was 0.852 (95% CI: 0.74-0.97) (Figure 2). The optimal cut-off point was 205.87 ng/mL with a sensitivity of 100% and a specificity of 54%. Both the curves were above the reference line which suggested that the curve predicted individuals with OSCC and OPMD. 

**Table 1:** Demographic and Clinicopathological Data of the Subjects in OSCC and OPMD Groups

Sl	Age	Gender	Site	Diagnosis
1.	63	Male	Retromolar trigone	Well-differentiated SCC
2.	55	Male	Alveolus – maxillary	Poorly differentiated SCC
3.	57	Male	Buccal mucosa	Verrucous carcinoma
4.	56	Female	Buccal mucosa	Poorly differentiated SCC
5.	48	Male	Buccal mucosa	Moderately differentiated SCC
6.	55	Male	Buccal mucosa and lip	Moderately differentiated SCC
7.	70	Female	Buccal mucosa	Moderately differentiated SCC
8.	44	Male	Retromolar trigone	Moderately differentiated SCC
9.	41	Male	Buccal mucosa	Well differentiated SCC
10.	58	Female	Buccal mucosa	Poorly differentiated SCC
11.	78	Female	Alveolus – mandible	Well-differentiated SCC
12.	71	Male	Retromolar trigone	Well-differentiated SCC
13.	40	Female	Buccal mucosa and Retromolar trigone	Moderately differentiated SCC
14.	60	Female	Lateral border of the tongue	Moderately differentiated SCC
15.	35	Female	Buccal mucosa	Moderately differentiated SCC
16.	68	Male	Floor of the mouth	Well-differentiated SCC
17.	56	Male	Lateral border of tongue	Poorly differentiated SCC
18.	63	Female	Floor of the mouth	Well-differentiated SCC
19.	78	Male	Buccal mucosa	Verrucous carcinoma
20.	47	Male	Buccal mucosa	Moderately differentiated SCC
21.	95	Female	Alveolus – Mandible	Moderately differentiated SCC
22.	35	Male	Buccal mucosa	Well differentiated SCC
23.	59	Male	Lateral border of tongue	Poorly differentiated SCC
24.	75	Female	Alveolus – Maxilla	Poorly differentiated SCC
25.	49	Male	Buccal mucosa	OSMF
26.	58	Male	Hard palate	Leukoplakia
27.	68	Male	Buccal mucosa	Erythroplakia
28.	26	Male	Lower lip	Leucoplakia
29.	27	Male	Buccal mucosa	OSMF
30.	49	Female	Buccal mucosa	Erythroplakia
31.	32	Male	Lower lip	Leukoplakia
32.	45	Male	Buccal mucosa	OSMF
33.	39	Female	Alveolus – Mandible	Leukoplakia
34.	26	Male	Retromolar trigone	Erythroplakia
35.	33	Female	Buccal mucosa	Erythroplakia
36.	44	Female	Buccal mucosa	OSMF
37.	79	Male	Buccal mucosa	Erythroplakia
38.	41	Female	Buccal mucosa	OSMF
39.	48	Male	Buccal mucosa	Leukoplakia
40.	62	Female	Buccal mucosa	Erythroplakia
41.	43	Male	Hard palate	Erythroplakia
42.	43	Male	Lateral border of the tongue	Erythroplakia
43.	33	Male	Buccal mucosa	OSMF
44.	35	Male	Buccal mucosa	OSMF

**Table 2:** Age and Gender Distribution among the Study Groups

	Age	Male	Female
	Mean±SD (Range)	N (%)	N (%)
OSCC	58.63±14.79 (35-95)	14 (58.3)	10 (41.7)
OPMD	44±14.19 (26-79)	14 (70)	6 (30)
Tobacco Habits	52.86±11.59 (31-71)	19 (86.4)	3 (13.6)
Controls	48.09±11.73 (23-69)	15 (68.2)	7 (31.8)
Total	51.23±14.06 (23-95)	62 (70.45)	26 (29.55)

**Table 3:** Comparison of Mean Salivary MMP-9 Concentration among the Study Groups

Group	Mean±SD	P-value	Post-hoc test
OSCC	387.87± 126.51	<0.001	OSCC, OPMD > TH, Controls
OPMD	339.86±115.70		
Tobacco Habits	227.75±54.13		
Controls	213.91±67.11		

**Table 4:** Mean Salivary MMP-9 Concentration with Respect to the Differentiation of OSCC and Different Conditions of OPMD

		Mean ±SD	P-value	Post-hoc test
OSCC	Well-differentiated	353.65±108.47	0.014	Poor> Moderate, Well
	Moderately differentiated	342.82±74.27		
	Poorly differentiated	506.78±134.37		
OPMD	Erythroplakia	406.28±154.23	0.128	-
	Leukoplakia	299.09±99.69		
	OSMF	293.08±39.58		

## Discussion

OSCC is the most prevalent form of malignancy in the oral cavity. It is often detected in advanced stages leading to lower survival rates. The prime reason is grading the lesion based on TNM staging criteria, which are highly subjective and open to error. (Wu et al., 2010) Unfortunately, two neoplasms with the same TNM classification can behave very differently and may have a heterogeneous response to similar kind of treatment. Hence, there is an ever-growing struggle to identify suitable biological indicators to establish the character and aggressiveness of a neoplasm. 

Few studies evaluated various tumor markers in the saliva of OSCC subjects (Ghallab and Shaker, 2017; Peisker et al., 2017; Lee et al., 2018). There is a need to identify specific and sensitive molecular biomarkers, which can be used to screen OSCC and OPMD. A systematic review enlisted eight useful biomarkers for detecting OSCC and OPMD (Hema Shree et al., 2019). They are mammary serine protease inhibitor, Ki67, phosphorylated-Src, carbonyls, 8-oxoguanine DNA glycosylase, Cyclin D1, metalloproteinase-9, and lactate dehydrogenase. MMPs can degrade the extracellular matrix. It is secreted by neutrophils, macrophages, and fibroblasts, on the stimulus provided by transforming growth factor β and interleukin8 (Venugopal and Uma Maheswari, 2016). The MMP sustains the bioavailability of growth factors, thus aiding in the proliferation of cancerous cells.

Our study demonstrated higher levels of saliva MMP-9 concentration in OSCC and OPMD subjects than subjects with tobacco habits and control groups. Previous studies Chen et al., (2008) and Ghallab and Shaker, (2017) reported higher levels of MMP-9 in OSCC and atrophic oral lichen planus than non-atrophic oral lichen planus. 

The optimal cut-off point for OSCC versus control was 214.37 ng/mL, which was similar to the previous study (Ghallab and Shaker, 2017) but higher than Peiskar et al., (2017). The optimal cut-off point for OPMD versus control was 205.87 ng/mL which was similar to the previous studies. 

Our study showed a significant difference in the mean MMP-9 with respect to the differentiation of OSCC. Kosunen et al. reported that there was a correlation between strong stromal MMP-9 staining intensity and poor differentiation of the cells (Kosunen et al., 2007).

This study showed higher levels of MMP-9 in subjects with tobacco habits; however, no significant difference was seen with the control group. Higher levels of MMP-9 have been found in people chewing gutkha regularly compared to controls (Javed et al., 2015). The difference could be due to the inclusion of subjects with all types of tobacco habits in our study. Tobacco, if consumed in various forms, causes surface changes and keratinization in mucosa which leads to alteration of MMP-9 secretion. This change in concentration can be traced in serum as well as the saliva of cancer subjects and subjects diagnosed with OPMD (Peisker et al., 2017).

The role of MMP-9 as a potential biomarker has been studied in various cancers (Huang, 2018). MMP-9 has been quantitatively measured in saliva and compared with the serum of OSCC and OPMD subjects with a significant positive correlation (Ghallab and Shaker, 2017). However, Mardani et al., (2014) reported a decreased level of serum MMP-9 in subjects having tumors of the salivary gland highlighting no significant role in the progression. 

MMPs can be classified as gelatinases, collagenases, stromelysins, and membrane-type MMPs (Wu et al., 2010; Hema Shree et al., 2019) due to their working mechanism which can lead to fragmentation of the FAS receptors, reduction of natural killer cells, and preventing apoptosis. They can have dual role in angiogenesis, increase the bioavailability of vascular endothelial growth factor receptor (VEGFR) (Venugopal and Uma Maheswari, 2016), active role in the celltocell adhesion, celltoextracellular matrix adhesion, breakdown of collagen (type IV, V, VII, X), fibronectin and elastin, (Chen et al., 2008) disintegrate several proteins, chemokines, cytokines, and alter their receptors to regulate cell growth and inflammation (Henriques et al., 2012).

In addition to oral clinical examination, screening by various imaging modalities and biopsy, salivary biomarker assay can be supplemented for early detection and progression of OPMD and OSCC. Developing a non-invasive saliva-based testing kit could be used as a new diagnostic adjunct for a community screening of subjects with OSCC and OPMD. 

In conclusion, to conclude, within the limits of this clinical study, salivary MMP-9 has a predictive role in the diagnosis of OSCC and OPMD. This study gives us an insight into the role of saliva MMP-9 as an essential adjunct salivary biomarker in the incidence and progression of OPMD into OSCC. It can be applied clinically as an early detection diagnostic tool in the future. Future studies on large populations should evaluate the possibility as an adjunct for diagnosis, during the treatment and follow-up.
